# Propiedades ópticas y mecánicas del zirconio translúcido como material restaurador óptimo en prótesis fija: Una revisión de la literatura

**DOI:** 10.21142/2523-2754-1003-2022-121

**Published:** 2022-09-28

**Authors:** Adrián Mauricio Santillán-Guerra, Viviana Maira Ticona-Orellana, Sergio Ricardo Escuza-González, Sergio Marcelo Delgado-Castillo, Wendy Isabel Huamán-Laredo, Katherine Joselyn Atoche-Socola, Carlos Aníbal Munive-Campos

**Affiliations:** 1 Carrera de Estomatologíade la Universidad Científica del Sur. Lima, Perú. amsg2502@gmail.com, 100044366@cientifica.edu.pe, sergioricardoeg@gmail.com, marcelodc776@gmail.com, wendyhuaman63@gmail.com. Universidad Científica del Sur Carrera de Estomatología Universidad Científica del Sur Lima Peru amsg2502@gmail.com 100044366@cientifica.edu.pe sergioricardoeg@gmail.com marcelodc776@gmail.com wendyhuaman63@gmail.com; 2 División de Rehabilitación Oral de la Universidad Científica del Sur. Lima, Perú. katoche@cientifica.edu.pe, cmunive@cientifica.edu.pe Universidad Científica del Sur División de Rehabilitación Oral Universidad Científica del Sur Lima Peru katoche@cientifica.edu.pe cmunive@cientifica.edu.pe

**Keywords:** zirconio traslúcido, prótesis fija, edentulismo parcial, estética dental, translucent zirconia, Fixed prosthesis, partially edentulous,dental aesthetics

## Abstract

**Introducción:**

La principal consecuencia de no tratar a tiempo la caries dental es la pérdida total o parcial del diente. Una de las alternativas para reemplazar la pieza dentaria perdida y devolver las funciones es el uso de prótesis fijas. Hoy en día existen 3 generaciones del policristal de zirconio estabilizado con itrio (Y-TZP). En la tercera generación, el contenido de itrio se incrementó hasta aproximadamente un 5% en moles (5Y), lo que estabilizó los materiales en aproximadamente un 50% de fase cúbica y un 50% de fase tetragonal, para obtener así un zirconio translúcido (5Y) con una alta estética y mayor naturalidad para la corona dental.

**Objetivo:**

Identificar las diferentes propiedades ópticas y mecánicas del zirconio translúcido frente a otros materiales restauradores utilizados en prótesis fija.

**Materiales y métodos:**

Se evaluaron 115 artículos científicos de las fuentes de información Medline (vía PubMed), Science Direct, Scopus, SciELO y LILACS, publicados en los últimos 5 años, tanto en español como en portugués e inglés. Se incluyeron 51 artículos que cumplieron con los criterios de selección; estos textos fueron ensayos clínicos aleatorizados y estudios observacionales, estudios in vitro y estudios in vivo que evaluaron las propiedades ópticas y mecánicas del zirconio translúcido como material óptimo restaurador en prótesis fija. Se excluyeron las revisiones sistemáticas, los informes de casos y los editoriales.

**Resultados:**

El zirconio, al ser un material relativamente nuevo y en evolución constante, cuenta con estudios en su mayoría in vitro, entre los que destacan los que hablan de sus propiedades mecánicas y ópticas, que varían según la generación a la que pertenece el material, así como de la adhesividad del material, la cual por ahora no es viable, por lo que resulta indispensable realizar más estudios sobre este material.

**Conclusiones:**

Podemos mencionar que el zirconio ha ido evolucionado a lo largo del tiempo, para proporcionar mejores propiedades ópticas, pero, como consecuencia, ha reducido su resistencia flexural y tenacidad a la fractura. Sin embargo, este material brinda una buena biocompatibilidad con las estructuras orales. Por otro lado, el zirconio translúcido es un material no grabable, por lo que se busca nuevos métodos para mejorar su adhesión.

## Abstract (en)

**Introduction:**

The main consequence of not treating tooth decay in time is the total or partial loss of the tooth. One of the alternatives to replace the lost tooth and return the functions is through fixed prostheses. Today there are 3 generations of yttrium-stabilized zirconium polycrystals (Y-TZP). Seeing that in the third Generation the yttrium content was increased to approximately 5% in moles (5Y), stabilizing the materials approximately 50% of cubic phase and 50% of tetragonal phase, thus giving a translucent zirconium (5Y) with a high aesthetics and greater naturalness to the dental crown.

**Objective:**

To identify the different optical and mechanical properties of translucent zirconium compared to other restorative materials used in fixed prostheses.

**Materials and methods:**

This narrative review evaluated 115 scientific articles from the Medline information sources (via PubMed), Science Direct, Scopus, SciELO and LILACS from the last 5 years, published in Spanish, Portuguese and English. We included 51 articles that met the selection criteria which were randomized clinical trials and observational studies, in vitro studies and in vivo studies that evaluated the optical and mechanical properties of translucent zirconium as an optimal restorative material in fixed prostheses. We excluded systematic reviews, case reports and editorials.

**Results:**

It was observed that zirconium being a relatively new material and in constant evolution has studies mostly in vitro, among which stand out those that speak both of the mechanical and optical properties that vary depending on the generation to which the material belongs, as well as the adhesiveness of the material which is for now not viable, therefore, it is essential that more studies be carried out on this material.

**Conclusions:**

We can mention that zirconium has evolved over time, providing better optical properties, but as a consequence it has been reducing flexural strength and fracture toughness. However, this material provides good biocompatibility with oral structures. On the other hand, translucent zirconium according to studies is a non-recordable material, so new methods are sought to improve its adhesion.

## INTRODUCCIÓN

La principal consecuencia de no tratar a tiempo la caries dental es la pérdida total o parcial de las estructuras que componen el diente, lo cual da como consecuencia la disminución en la funcionalidad y estética de nuestro aparato estomatognático ^1^. Una de las alternativas para reemplazar la pieza dentaria perdida y devolver las funciones es el uso de prótesis fijas, las cuales pueden ser metálicas o de porcelana, como disilicato, ceromero y zirconio. Uno de los materiales actualmente usado y con buenas propiedades ópticas y mecánicas es el zirconio ^2^, debido a que guarda una gran semejanza con el diente natural ^3^.

Hoy en día, existen 3 generaciones de policristal del zirconio estabilizado con itrio (Y-TZP). En la primera generación, las zirconias utilizadas en odontología, en su mayoría, contenían 3 mol% de itria (3Y) y 0,25% de peso de alúmina, material conocido como zirconio opaco. Este proporciona una resistencia significativamente mayor y está indicado para restauraciones posteriores de la boca ^4^^,^^5^. En la segunda generación, se presentó una reducción de la concentración de alúmina y se realizó la sinterización a una alta temperatura del 3 Y, para lograr menor opacidad; sin embargo, este material carece de translucidez. En la tercera generación, el contenido de itrio se incrementó hasta aproximadamente un 5% en moles (5Y), lo que estabilizó los materiales, aproximadamente, un 50% en fase cúbica y un 50% en fase tetragonal ^5^^,^^6^, y se obtuvo como resultado un zirconio translúcido (5Y), con una alta estética y mayor naturalidad para la corona ^5^.

En cambio, la gran cantidad de estas partículas genera una baja resistencia en comparación con el zirconio 3% mol de itria (3Y) ^7^^,^^8^. Las condiciones de la sinterización del material influyen mucho en su translucidez, ya que el tamaño y la transmisión de las partículas de zirconio pueden aumentar o disminuir. Mientras existan más partículas isotrópicas, mayor será esta característica óptica ^7^^,^^9^. Por el contrario, sus características mecánicas serán más susceptibles ^9^. La zirconia translúcida presenta características ópticas aceptables, pero por debajo de las del disilicato de litio y el esmalte dental, según los parámetros de translucidez, los cuales están entre 11,2 y 15,3 unidades, mientras que el disilicato de litio cuenta con valores de 16,89 unidades ^10^.

Algunos estudios señalan que el zirconio está surgiendo como una alternativa prometedora a sistemas de implantes convencionales a base de titanio para la rehabilitación oral, con mejores propiedades biológicas, estéticas, mecánicas y ópticas ^11^. También se han perfeccionado zirconias translúcidas con una mejor estética que las zirconias de alta resistencia, al reducir la opacidad. Sin embargo, los estudios sobre su translucidez y resistencia son muy escasos. El disilicato de litio era el material más translúcido y, sin embargo, el más débil, mientras que la zirconia de alta resistencia era la cerámica más opaca y resistente ^12^.

El propósito de la presente investigación es identificar las diferentes propiedades ópticas y mecánicas del zirconio translúcido frente a otros materiales restauradores utilizados en prótesis, para verificar si cumplen los planteamientos y, luego, concluir si el zirconio se adapta adecuadamente y cumple los requisitos para establecerse como el material *gold standard*.

## MATERIALES Y MÉTODOS

La búsqueda se realizó en las siguientes bases de datos: Medline (vía PubMed), Science Direct, Scopus, SciELO y LILACS. Se buscó trabajos publicados en los últimos 5 años, tanto en español, portugués e inglés. Se usaron las siguientes palabras claves: *zirconio*, *zirconio translúcido*, *prótesis fija*, *propiedades mecánicas* y *propiedades ópticas*. Al finalizar la búsqueda, se obtuvo un resultado de 115 publicaciones; luego de una selección por título y resúmenes, solo fueron utilizados para esta revisión 51 artículos, que cumplieron los criterios de selección que incluían ensayos clínicos aleatorizados y estudios observacionales. Estos trabajos evalúan las propiedades ópticas y mecánicas del zirconio translúcido como material óptimo para la restauración con prótesis fija. Se excluyeron las revisiones de literatura, las revisiones sistemáticas, los reportes de casos y los editoriales.

## RESULTADOS Y DISCUSIÓN

### Generaciones del zirconio

La zirconia hoy en día está siendo usada por más odontólogos en tratamientos como carillas, coronas, estructuras y prótesis sobre implantes. Este material presenta 4 generaciones bien marcadas, las cuales, según su proceso, tienen diferentes indicaciones de uso. La primera fue la zirconia policristalina estabilizada con itrio (3Y-TZP), que cuenta con unas excelentes propiedades mecánicas, pero pobres propiedades ópticas. A partir de este material se desarrollaron otros 3: la segunda generación de 3Y-TZP, la tercera generación de 5Y-TZP y la cuarta generación de 4Y-TZP ^13^. Una de las orientaciones fue a la reducción del tamaño del grano del material, ya que, mientras más pequeño, se obtiene mayor translucidez y, a su vez, mejores propiedades ópticas. En cambio, unos de los grandes retos es cuidar sus propiedades mecánicas, debido a que la disminución o aumento de los granos del material afecta la resistencia del material, puesto que no sufre cambios la zirconia cúbica ^5^^,^^14^. 

### Primera generación

Esta generación de la zirconia pasa por un proceso de sinterización a una temperatura de 1600 °C, debido a lo cual tiene una disminución de la resistencia a la flexión, ya que su comportamiento es negativo con respecto a la resistencia y más particularmente a la estabilidad a largo plazo. Esta generación monolítica de zirconio no logró estabilizarse ^15^.

Hay que mencionar que la ventaja del 3Y-TZP de primera generación es que puede reaccionar positivamente a la formación de grietas con endurecimiento por transformación. Este endurecimiento le proporciona su alta tenacidad a la fractura; sin embargo, la desventaja que presenta es la opacidad, debido a la presencia de alúmina. Este material se le agregó para ayudar a prevenir la formación de poros y estabilizar el zirconio tetragonal ^4^^,^^6^^,^^16^.

### Segunda generación

En el 3Y-TZP, el contenido de alúmina en peso se redujo del 0,25% al 0,05%, lo que le proporcionó mayor translucidez ^16^ y mejor transmisión de luz, así como mayor estabilidad a largo plazo y muy buena resistencia. Estudios *in vitro* demostraron que esta generación tiene una mayor translucidez y resistencia ^15^; sin embargo, es más susceptible a la degradación a baja temperatura; debido a que posee menos alúmina para estabilizar la fase tetragonal ^4^^,^^6^^,^^16^. 

### Tercera generación

La tercera interacción del zirconio dental es metaestable en la fase tetragonal y contiene una proporción de fase cúbica de hasta un 53%. Logra ser totalmente estabilizado con una mezcla cúbica (estructura tetragonal). Los cristales cúbicos tienen un volumen mayor en comparación con los tetragonales; esto significa que la luz se dispersa con menos fuerza en los límites del grano y las porosidades residuales, con lo que gana translucidez ^15^. Sin embargo, la zirconia cúbica estabilizada no se transforma a temperatura ambiente y, debido a esto, no sufre procesos de endurecimiento por transformación ni degradación a baja temperatura. En resumen, tiene una baja propiedad mecánica y mejores propiedades ópticas ^(6^^,^^16^. 

### Cuarta generación

En 2017 se introdujo esta cuarta generación de zirconio, con un contenido del 4% moles de itrio en comparación con la tercera generación, lo que produjo una reducción de las propiedades ópticas y un incremento de las propiedades mecánicas ^17^, gracias a una reducción de la fase cúbica de alrededor del 30% ^4^^,^^16^. El resultado fue un material más fuerte que el 5Y-TZP, pero menos traslúcido ^16^ (Tabla 1).

**Tabla 1:** Fórmulas, año y resumen de las propiedades mecánicas y ópticas de cada generación del zirconio

	Generaciones del zirconio			
	Primera generación	Segunda generación	Tercera generación	Cuarta generación
Fórmula	3Y-TZP	3Y-TZP	5Y-TZP	4Y·TZP
Mecánica	+++	++	.	+
Óptica	.	+	+++	++
Año	1995	2013	2015	2017

Interpretación: Mejores +++ Mejor ++ Buena + Peor -

### Elección del buen material protésico

Para elegir el material protésico, la armonía funcional entre factores mecánicos y ópticos (asociado a la estética), crea competencia por asociar ambos propósitos para que sean proporcionales positivamente, pues existe evidencia que la apariencia translúcida del material afecta negativamente su resistencia ^15^. Con el tiempo, se han logrado mejorar las propiedades ópticas de los materiales cerámicos; sin embargo, el éxito clínico a largo plazo está mayormente ligado a una adecuada selección, una buena preparación dentaria, fabricación, segmentación y control oclusal para poder obtener una adecuada longevidad ^17^ (Tabla 2).

**Tabla 2:** Puntos por considerar para elegir un buen material protésico

Elección de un buen material protésico
Biocompatibilidad con estructuras dentales y
periodontales
Integración estética
Conservación de la estructura dental remanente
interacción mecánica óptima

#### Biocompatibilidad con estructuras dentales y periodontales

Las restauraciones directas e indirectas, sobre contorneadas, guardan una relación estrecha con las enfermedades gingivales, ya que pueden ocasionar mayor pérdida ósea interproximal y profundidad de sondaje. Además, es un factor que ayuda a la acumulación de biofilm, lo que trae como consecuencia un aumento de la microflora subgingival y ocasiona enfermedades periodontales y microfiltraciones que forman caries recurrentes y, finalmente, un fracaso de la prótesis fija ^18^^,^^19^.

Las diferentes características de los materiales dentales, como su superficie rugosa e irregular, su energía superficial y su ubicación en relación con la encía influyen en el estado del tejido periodontal y la colonización bacteriana. Por otro lado, el estado de salud puede verse afectado si el paciente presenta reacciones alérgicas, trastornos galvánicos, efectos tóxicos y cambios en el organismo ^18^^,^^20^^-^^26^.

#### Integración estética

Para lograr el color, la translucidez y el grosor adecuados en la restauración se deben considerar ciertos factores en los procesos de manufacturación como en los procedimientos clínicos ^26^. Factores extrínsecos, como la capa del cemento, e intrínsecos, como la estructura del material. Para lograr la integración estética, se deben considerar, además de estos factores, la elaboración del material según la marca y la resistencia asociada con la translucidez, es decir, qué tanto se integran para lograr la mayor estética posible en la restauración ^27^.

El grosor de la restauración desempeña una tarea muy importante, pues está relacionado con los otros materiales, como los fondos y el cemento. Si ambos componentes son de color similar al del diente natural, el grosor de la zirconia puede mantener niveles normales; por ello, se recomienda armonizar los colores cemento-cerámica ^26^. Por ejemplo, la zirconia de alta translucidez es recomendable en restauraciones anteriores ^27^.

#### Conservación de la estructura dental remanente

Toda vez que un paciente parcialmente desdentado consulta sobre la posibilidad de reponer sus piezas dentales mediante una prótesis fija, cobrarán gran importancia la resistencia y la capacidad adhesiva del material restaurador, con el fin de conservar al máximo la estructura dental ^28^^,^^29^.

#### Interacción mecánica óptima

Es importante saber que las cerámicas utilizadas en la odontología se caracterizan por la tenacidad a la fractura y la resistencia a la flexión, puntos necesarios para obtener un buen desempeño clínico. 

La tenacidad a la fractura es proporcionada principalmente por endurecimiento mediante transformación, y se encuentra en el rango de 4 a 8 MPa√m. Esto dependerá del tamaño del grano, la temperatura de sinterización y el contenido de litio ^30^. Por tal motivo, si el tamaño del grano es muy pequeño, puede repercutir negativamente el proceso de mecanismo de endurecimiento por transformación, como consecuencia, hay una reducción de la fuerza y la resistencia a la fractura ^31^. La resistencia a la flexión es una propiedad importante y se considera como el potencial clínico frente a la restauración de cerámica dental, ya que es un método relevante y confiable para evaluar la durabilidad del material cerámico, pues permite restauraciones con menos susceptibilidad a la fractura ^6^ (Tabla 2).

Los clínicos tienen la misión de brindarle al paciente un tratamiento exitoso y eso se logra trabajando con materiales que proporcionen una adecuada función y longevidad, así como una gran biocompatibilidad con las estructuras dentarias, para disminuir el riesgo de contraer enfermedades gingivales que ocasionen la pérdida ósea interproximal y enfermedad periodontal ^17^^-^^19^. La integridad estética, la conservación de la estructura dental remanente y la interacción mecánica son puntos importantes, ya que proporcionan una restauración más natural, resistente y conservadora ^26^^,^^28^^-^^31^.

### Importancia de la transmisión lumínica del zirconio translúcido

Actualmente, los pacientes quieren un acabado natural y muy estético, lo que se está logrando mediante modificaciones estructurales, aumentando el contenido total de la fase cúbica y reduciendo el grano, tamaño, distribución de defectos y porosidad para obtener una mejor translucidez de la zirconia ^32^^,^^33^. También es importante mencionar que, en la estética de las restauraciones con cerámica dental, el color, la translucidez, la textura de la superficie y la forma; por lo tanto, la estabilidad del color y la translucidez, son parámetros de suma importancia para el éxito ^34^^-^^37^. 

#### Sinterización, una fase importante para llegar al color y la translucidez ideal

El color de las restauraciones con cerámica puede verse afectado por el tiempo y el grado de sinterización ^32^^-^^35^. En un estudio sobre la sinterización de la zirconia, se examinaron el efecto de la temperatura del proceso de sinterización, así como los procesos de condición atmosférica durante el procedimiento de sinterización y los métodos de calentamiento ^35^^,^^36^. Se reportó que el aumento de temperatura en el proceso de sinterización aumentó la translucidez ^36^ y el tamaño del grano, pero no cambió la concentración de la fase cristalina ^37^. La temperatura de sinterización, de 1600 °C, produjo un cambio en la translucidez ^38^ y el proceso de enfriamiento rápido produjo una fase tetragonal, que aumentó esa propiedad ^39^. Es importante mencionar que, al aumentar la temperatura de sinterización, se reduce la porosidad, se incrementa la densidad y, en consecuencia, se produce menos dispersión de luz y más transmisión de esta ^36^^-^^39^. 

Un estudio reciente analizó 2 tipos de zirconias monolíticas translúcidas precoloreada y presinterizada (Katana - KAT; Kuraray Noritake Inc. y NexxZr - NEX; Sagemax Bioceramics, Cía.). Ambas fueron afectadas por el cambio de tiempo de sinterización y dicho tiempo fue similar en los dos casos (1 o 3 horas); sin embargo, para NEX, este efecto dio como resultado mayor diferencia de color respecto del color de las muestras cuando se empleó el tiempo especificado por el fabricante. La sinterización de 1 hora aumentó la translucidez del KAT. De acuerdo con la translucidez deseada, el tiempo de sinterización de la zirconia KAT puede modificarse con respecto al indicado por el fabricante, sin efectos relevantes en el color ^35^. 

En otro estudio se menciona que el espesor es también un factor importante, debido a que la disminución de este demostró tener una mayor translucidez para los 5Y-TZP. Cabe recalcar que esta todavía no es igual a la translucidez del esmalte. Además, una disminución de ZrO2 y un espesor de menos de 1,5 mm pueden estar contraindicados, sobre todo en el área oclusal, ya que tiene propiedades mecánicas más bajas que 3Y-TZP, lo que lo hace más susceptible a la rotura ^32^.

#### La eficiencia del acabado y el pulido en el color de una restauración

Los cambios de color y las propiedades de la superficie son factores imprescindibles al momento de hacer la restauración. Es necesario realizar el pulido debido a que cualquier rugosidad en la superficie podría mancharse con las bebidas, por ejemplo, el café ^40^^,^^41^.

Un artículo menciona que el color y la translucidez de la zirconia translúcida pueden verse afectados por la marca de la zirconia y el protocolo de sinterización, y agrega que el acabado de la superficie y el termociclado del café también afectan el color y la translucidez, pero son aceptables clínicamente ^42^.

La disminución en el tamaño del grano, el tiempo y el grado de sinterización proporciona una translucidez ideal y un cambio de color más estético ^32^^-^^35^. Además, un estudio muestra que el tiempo de sinterización varía según la marca del zirconio translúcido ^34^. Otro punto por mencionar es que el espesor constituye otro factor importante para obtener una mejor translucidez, junto con el acabado y pulido ^31^^,^^40^^-^^42^.

### Relevancia de la transferencia de fuerza a través del zirconio

Con respecto a las cerámicas a base de óxidos de zirconio, específicamente el 3Y-TZP o zirconia, como material restaurativo, este ha generado interés en la comunidad científica debido a que sus propiedades mecánicas son las mejores jamás reportadas para cualquier otra cerámica dental. Esto se relaciona con su tamaño de partícula pequeña y la expansión que acompaña la transformación de fase frente a una grieta en propagación. Entre los materiales para restauraciones libres de metal, la zirconia exhibe los mayores valores de resistencia flexural (800-1300 MPa), la resistencia a la fractura es mayor a 5,0 MPa x m0,5 (6-8 MPa x m0,5), el módulo elástico resiste hasta 350 GPa en el dióxido de zirconio reforzado con alúmina. En este caso, existe menor contracción por sinterización (8-11%), cuando el procedimiento de construcción de la estructura es manual, comparado con la zirconia presinterizada maquinada (cercana al 25%, cuando la sinterización ocurre a muy altas temperaturas), lo que puede explicar valores menores de propiedades mecánicas del In Ceram Zirconia® respecto de la zirconia sin refuerzo con alúmina. Las propiedades mecánicas de la zirconia dependen del tamaño de su partícula ^28^.

Las formulaciones con tamaños de partícula muy grandes son menos estables y más susceptibles a la transformación espontánea de fase t-m, mientras que tamaños de partícula más pequeños (<1 µm) están asociados con una tasa de transformación menor. Sin embargo, con partículas por debajo de 0,2 µm, la transformación no es posible, lo que produce una reducción de la resistencia a la fractura ^29^.

Hoy en día, la zirconia translúcida está siendo muy estudiada, ya que su resistencia y tenacidad han podido ser afectadas como consecuencia del aumento del contenido cúbico del material ^43^. La zirconia translúcida presenta más resistencia a la fractura que el disilicato de litio, pues presenta 0,5 mm de espesor comparado con los 1,5 mm del otro material ^10^. Pese a ello, el zirconio convencional (3Y) presenta mayor resistencia de fractura que su generación translúcida (5Y) y cuenta con el mismo módulo elástico (200-210 GPa), que es más elevado que el de disilicato de litio (95-105 Gpa) ^47^.

### Comparación funcional del zirconio translúcido frente a otros materiales de última generación

El óxido de zirconio translúcido, presenta una disminución de los procesos inflamatorios periodontales, debido a la escasa formación de biofilm dental. Confiere una mejor adaptación marginal, lo que evita infiltraciones bacterianas que producen caries recurrentes. De esta forma, protege los sustratos dentales y ofrece una excelente biocompatibilidad con las mucosas y los tejidos de la cavidad bucal ^20^^-^^24^. Estudios que comparan el zirconio con las coronas metal cerámica, ante la caries recurrente y la enfermedad periodontal, han obtenido un incremento de los valores, el índice de sangrado y la profundidad al sondaje de las coronas convencionales de metal cerámica ^22^^,^^25^.

Con el fin de darle una característica translúcida óptima, se fabricaron generaciones posteriores a la 3Y-TZP, lo que reduce en gran medida la concentración de aluminio y aumenta el grado de sinterización y el aumento de contenido de itria, que es proporcional a la cantidad de circonio cúbico ^43^. Algunos autores indican que el tipo del material, como el sustrato y el cemento, pueden influenciar en la translucidez ^6^. Se realizó un estudio de sinterización convencional y rápida a dos tipos de zirconio el 3Y-TZP y al zirconio del 5% de moles 5 Y-TZP, y se concluyó que este último es más susceptible dándole una coloración óptima. Una investigación *in vitro*^44^ mostró que la zirconia cúbica ultra translúcida y la super translúcida presentaron una mayor translucidez en comparación con el disilicato de litio. No obstante, otro estudio *in vitro*^45^ concluyó que la zirconia altamente translúcida tiene mayor translucidez que el 3Y-TZP, aunque el disilicato de litio, por la forma de crecimiento de sus cristales, presenta mayores propiedades translúcidas. 

Por otro lado, el aumento de cristales cúbicos tiene un impacto negativo en las propiedades mecánicas de la zirconia, por lo que, con la evolución del material, se han visto disminuidas características del material como su fuerza flexural y la resistencia a fracturas ^17^. Un estudio que comparó 2 tipos de zirconio y una marca de disilicato de litio explican que el zirconio translúcido tiene una fuerza flexural comparable a la de una restauración de cerámica, y como resultado no existió una diferencia significativa entre el zirconio translúcido y el disilicato de litio ^12^. Otro estudio *in vitro* sobre las propiedades del zirconio translúcido (5Y-TZP) en comparación con las del disilicato de litio demostró que el primero sí tiene una mayor fuerza flexural y resistencia a las fracturas ^46^. El zirconio es, en realidad, un material bastante versátil y sus diferentes generaciones pueden adecuarse a distintas necesidades.

Diversas investigaciones han tratado de identificar cómo afectan el tipo, el grosor del material, los colores del sustrato y el cemento a la translucidez y el color, mediante la evaluación, principalmente, de las características que tienen el zirconio de alta translucidez y el disilicato de litio frente a estas variables. Se concluye que estas variables influencian el color y la translucidez de estos materiales ^2^^,^^3^^,^^43^. Por otro lado, algunos autores, señalan que la zirconia translúcida presenta mejores propiedades ópticas que el disilicato de litio; sin embargo, otros trabajos, que tuvieron como objetivo comparar parámetros ópticos, mecánicos y adhesivos entre el zirconio translúcido, el convencional y el disilicato de litio, concluyeron que el zirconio translúcido presenta una resistencia a la flexión mayor que del zirconio convencional, pero no mayor que la del disilicato de litio ^44^^,^^45^.

### Adhesividad

Respecto de la adhesividad, existen estudios sobre algunos tipos de materiales utilizados para determinar la unión entre resinas y cerámicos de alta resistencia. Se obtuvo una referencia en la que se controlaron 193 coronas unidas a pilares de zirconio mediante resina y logro el éxito en el 96,4% ^47^. Sin embargo, Scaminaci utilizó un proceso de envejecimiento de ambos materiales por almacenamiento de agua y demostró que la fuerza de unión del cemento y el material cerámico va degradándose ^48^.

También se realizaron estudios con láser de fibra para lograr una mejor adhesión en las prótesis de zirconio, analizando y comparando los resultados positivos con los posibles efectos secundarios, pero no se hallaron diferencias significativas en adhesión en ninguno de los dos grupos de estudio ^49^. Otro estudio, que aplicó láser Er: YAG para potenciar las condiciones de adhesión, tampoco mostró beneficios en la unión del zirconio con la resina ^50^.

En otra investigación se utilizó el método de termociclado para mejorar la adhesividad en tres grupos separados; el primero sin tratamiento y sistema adhesivo, el segundo utilizando un chorro con óxido de aluminio y sistema adhesivo, y el último con tratamiento triboquímico y sistema adhesivo. El estudio se llevó a cabo en grupos aleatorizados de zirconio con un tipo de tratamiento superficial. Los primeros resultados arrojan una mayor fuerza adhesiva en los grupos 1 y 3; sin embargo, se marca una diferencia clara entre ambos grupos luego del ciclo térmico, tras lo cual el grupo 3 que presentó mayor fuerza de unión y el 93% del peso de la muestra correspondía al zirconio ^51^.

Lo investigado con relación a la propiedad adhesiva del zirconio con otros materiales, como el fluoruro diamino o resina, se determinaron con resultados positivos funcionales en tasa de éxitos de aproximadamente 95 a 100 por ciento en dos estudios realizados previamente y tomados como referencia. Al contrastar las posibilidades de uso clínico, la degradación de la unión del material con el zirconio puede empezar a aumentar mediante el almacenamiento progresivo del agua debido a que ambos elementos comienzan y continúan un proceso acelerado de envejecimiento ^47^^,^^48^. Paralelamente, en busca de mejores posibilidades para la efectividad adhesiva, se plantean metodologías alternativas, como el uso de láseres para potenciar y reducir los efectos secundarios en las características finales que debe tener una restauración protésica a base de zirconio ^49^^,^^50^.

## CONCLUSIONES

La zirconia ha ido cambiando de generación en generación, tanto en su tamaño de grano como en su tamaño de itria y tiempo de sinterización, lo que proporciona propiedades ópticas de alta estética. Sin embargo, como consecuencia de este proceso, se puede producir una reducción de la resistencia flexural y la tenacidad a la fractura. Además, el zirconio translúcido posee una buena biocompatibilidad con las estructuras dentales y periodontales. Pero una de sus más grandes desventajas es que es un material ácido-resistente, por lo que se busca mejorar su adhesión con el uso de la abrasión con láser o con aire y partículas de aluminio, aunque esto no resulta muy viable. A pesar de los intentos experimentales por mejorar la capacidad de la adhesión del zirconio a partir del grabado con ácidos o láseres, los estudios referenciados indican que no existen mejoras en la adhesión, es por ello que se concluye que el zirconio translúcido no necesita ningún método grabador para potenciar su adhesividad. Por otro lado, el zirconio translúcido posee buenas propiedades mecánicas y ópticas; no obstante, la mayoría de los estudios que lo evidencian son *in vitro*, lo cual hace que sea difícil extrapolar ese resultado a la práctica odontológica.

## Financiamiento:

El siguiente proyecto no cuenta con ninguna beca específica de agencias del sector público, comercial o sin ánimo de lucro.
